# Gut microbiome associated with low anterior resection syndrome after rectal cancer surgery

**DOI:** 10.1038/s41598-023-34557-2

**Published:** 2023-05-26

**Authors:** Min Jung Kim, Soyoung Park, Ji Won Park, Jinsun Choi, Hyo Jun Kim, Han-Ki Lim, Seung-Bum Ryoo, Kyu Joo Park, Yosep Ji, Seung-Yong Jeong

**Affiliations:** 1 Department of Surgery, Seoul National College of Medicine, Seoul, Republic of Korea; 2 Colorectal Cancer Center, Seoul National University Cancer Hospital, Seoul, Republic of Korea; 3 Cancer Research Institute, Seoul National University, Seoul, Republic of Korea; 4 Bioinformatics Center, HEMpharma, Suwon-si, Gyeonggi-do Republic of Korea

**Keywords:** Digestive signs and symptoms, Dysbiosis, Rectal cancer, Clinical microbiology

## Abstract

This study aimed to assess the likely association of gut microbiome with low anterior resection syndrome (LARS) symptoms. Postoperative stool samples from patients with minor or major LARS after sphincter-preserving surgery (SPS) for rectal cancer were collected and analyzed using 16S ribosomal RNA sequencing method. The symptom patterns of LARS were classified into two groups (*PC1LARS, PC2LARS*) using principal component analysis. The dichotomized sum of questionnaire items (*sub1LARS, sub2LARS*) was used to group patients according to the main symptoms. According to microbial diversity, enterotype, and taxa, *PC1LARS* and *sub1LARS* were associated with frequency-dominant LARS symptoms and patients, while *PC2LARS* and *sub2LARS* were grouped as incontinence-dominant LARS symptoms and patients. *Butyricicoccus* levels decreased while overall LARS scores increased. The α-diversity richness index Chao1 showed a significantly negative correlation in *sub1LARS* and a positive correlation in *sub2LARS*. In *sub1LARS*, the severe group showed a lower *Prevotellaceae* enterotype and higher *Bacteroidaceae* enterotype than the mild group. *Subdoligranulum* and *Flavonifractor* showed a negative and a positive correlation with *PC1LARS*, respectively, while showing a negative relationship with *PC2LARS*. *Lactobacillus* and *Bifidobacterium* were negatively correlated to *PC1LARS*. Frequency-dominant LARS had decreased diversity of gut microbiome and showed lower levels of lactic acid-producing bacteria.

## Introduction

In 2020, 732,210 new cases of rectal cancer were reported, and 339,022 patients died of rectal cancer worldwide^1^. Colorectal cancer (CRC) was the fourth most prevalent cancer in South Korea in 2019, following thyroid, lung, and stomach cancer^2^. Multimodal treatments such as total mesorectal excision (TME), radiotherapy, or chemotherapy contribute to improved survival of rectal cancer patients. Thus, the number of long-term survivors has increased, and 170,504 patients in South Korea have survived for > 5 years after CRC diagnosis^2^. Similarly, recent advances have enabled > 70%–80% of rectal cancer patients to undergo sphincter-preserving surgery (SPS) with long-term survival^3,4^.

Up to 90% of rectal cancer patients undergoing SPS will develop subsequent bowel habit changes with varying severity, including urgency, fecal incontinence, and evacuation difficulty or incomplete emptying, termed low anterior resection syndrome (LARS)^5^. The quality of life (QOL) of rectal cancer survivors is closely associated to LARS severity^6–8^. Previously, it was believed that these functional problems could improve over time, but these problems have been reported to persist with LARS and so is its impact on patients’ QOL even after ≥ 5 years^9^.

LARS is considered a multifactorial syndrome, but its underlying pathophysiology has not been fully described^10^. The proposed pathophysiologic mechanisms include dysfunction of the internal anal sphincter, loss of anal canal sensation, absence of the rectoanal inhibitory reflex, local reflex disruption between the anus and the neorectum, and decrease in rectal reservoir capacity and compliance^11^. Clinical risk factors predicting the occurrence and severity of LARS have been extensively studied to prevent and treat LARS^12^. Nevertheless, a previous study conducted by UK researchers failed to prevent LARS by means of several clinical risk factors, such as age, sex, TME, tumor height, stoma, and preoperative radiotherapy^13^.

Only a few specific treatments with long-term effects have been reported^11^. To modulate postoperative bowel function, modifiable factors affecting bowel movements need to be investigated, and one of the modifiable factors associated with bowel function is the gut microbiome^14–16^. The gut microbiome affects colon motility and can be modified by diet, probiotics, and medications such as antibiotics^17–21^. Several studies have reported the likely association between gastrointestinal (GI) diseases and the gut microbiome in patients with irritable bowel syndrome^22–24^, inflammatory bowel disease^25^, and antibiotic-related *Clostridium difficile* colitis^26,27^. However, current research on the gut microbiome of LARS patients is scarce. Still, a previous report demonstrated that administering probiotics to LARS patients failed to improve LARS symptoms, but it positively modified serum immune markers^16^. Moreover, the association between the gut microbiome and LARS symptoms has not been studied. Understanding the gut microbiome of patients with LARS might provide a clue to the potential treatment of LARS patients with probiotics and/or prebiotics. Therefore, this study aimed to identify the gut microbiome of patients with LARS according to the main symptom groups and to compare the functional taxonomic differences between the groups.

## Methods

### Ethical approval of the study and informed consent

This was a retrospective cohort study based on the CRC cohort at Seoul National University Hospital. This cohort included patients who underwent surgery for CRC since 2014. The study design was approved by the Institutional Review Board (IRB) of the Seoul National University Hospital (IRB no. 1408-127-607). We followed the principles outlined in the Declaration of Helsinki. Informed consents were obtained from the study participants as the IRB approved.

### Fecal sample collection from LARS patients

In our cohort for CRC patients, fecal samples were collected prospectively from patients between September 1, 2017, and May 31, 2019, after an average of 13.3 and 2.2 months following LAR and ileostomy repair, respectively. The stool samples were frozen and stored at − 20 °C. The fecal samples included in this study were collected from patients who underwent SPS for CRC from September 2013 to March 2019 and had minor or major LARS postoperatively (LARS score > 20). We included patients who experienced postoperative complications, and those with diverting stomas were excluded from the analysis. Surgeries were performed by surgeons in a single tertiary center where more than 500 colorectal cancer surgeries per year are performed in a standardized manner.

### 16S bacterial rRNA microbiota analysis

Whole DNA in fecal samples was extracted using the Mag-Bind Universal Pathogen 96 Kit (Omega Bio-Tek, Norcross, GA, USA) with a Hamilton Microlab STAR liquid handler (Hamilton Laboratory Solutions, Manitowoc, WI, USA) after bead-beating the samples with the TissueLyser (Qiagen, Hilden, Germany), followed by amplicon PCR targeting the V3 to V4 region of the 16S bacterial rRNA gene using 341F and 805R primers (341F-CCTACGGGNGGCWGCAG, 805R-GACTACHVGGGTATCTAATCC). After DNA library preparation, indexing and quality checks were performed using the Nextera XT index kit (Illumina, San Diego, CA, USA) and Qubit4.0 (Thermofisher, Wilmington, DE, USA), and 300× two paired-end sequencing was performed using the MiSeq system (Illumina, San Diego, CA, USA). The average read depth of the raw FASTQ files was over 50,000 counts. The denoised amplicon sequence variant (ASV) features acquired after DADA2 in QIIME2 2020.2 (qiime dada2) were classified at the taxonomic level by a pre-trained classifier with Silva138^28–31^. The sequenced raw data have been submitted in NCBI Sequence Read Archive with BioProject accession number PRJNA882613.

### Definitions regarding LARS

The LARS questionnaire developed by Emmertsen et al. was used to assess postoperative bowel dysfunction following SPS^32^. Questionnaires were administered after an average of 13.3- and 2.2-months following LAR and ileostomy repair, respectively. The questionnaire contained five items: flatus incontinence, liquid stool incontinence, frequency, clustering, and urgency. Each item comprised three severity-weighted options. The LARS score was categorized into three groups: no LARS (0–20 points), minor LARS (21–29 points), or major LARS (30–42 points).

### Grouping LARS using principal component analysis (PCA) and sum of scores of questionnaire items

Patterns with minor and major LARS were grouped using an approach with PCA performed on the five LARS questionnaire items. After extracting the principal components (PCs), varimax rotation was used to create a structure with independent variables and high potential for interpretability. The minimum eigenvalues of 1.0, screen plot, and interpretability of the factors were used to determine the variables that should be retained in the LARS patterns. As we did in previous studies, the symptoms of patients with LARS were clustered into two groups: incontinence-dominant and frequency-dominant^33^.

In addition to this PCA-based score, another subgroup scoring and classification were introduced to generalize the two dominant symptoms, as the PCA score is unique to this study's dataset.

### Statistical and clustering analysis with microbiota data

The ASV feature table and taxonomic classification outputs were further processed using the phyloseq package in R to perform the diversity and taxonomic profiling analyses^34^. The total count normalization was performed with total sum scaling (known as relative abundance) without any ASV filtering step, except alpha-diversity richness inference, which uses singleton and doubleton count values. MaAsLin2 was used for taxonomic analysis at the genus level with continuous and categorical LARS-associated variables in this study after filtering taxa containing many zeros (zero in more than 90% of samples)^35^. Enterotype clustering was conducted using the Dirichlet multinomial package in R as previously described^36,37^. Statistical analyses were performed using nonparametric tests [Spearman’s correlation, Wilcoxon rank-sum test, and permutational multivariate ANOVA (PERMANOVA)] and generalized linear models, including MaAsLin2 in R version 4.1.1.

## Results

### LARS-associated symptom subgroups

Among 109 patients, 91 were classified into major LARS and 18 into minor LARS. The baseline characteristics of patients were shown in Supplementary Table S1. The mean age was 62.2 years, and 101 (92.7%) of patients were males. The mean tumor location from the anal verge was 7 cm, and 58.7% of patients had diverting ileostomy followed by ileostomy reversal. Preoperative or postoperative radiotherapy was administered in 55 (50.5%) patients, and 3 (2.8%) patients experienced anastomotic leakage.

**Figure 1:**
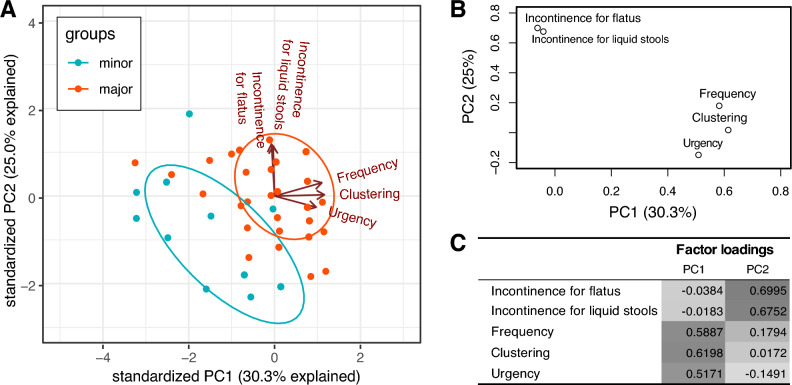
Principal component analysis (PCA) using low anterior resection syndrome (LARS) score from 109 patients. (**A**) PCA scores biplot, (**B**) loading plot of PCA on LARS questionnaire items, (**C**) factor loadings for each principal component (PC). It explains that there is 55.3% of total data variance with two principal components.

The questionnaire items for frequency, clustering, and urgency were grouped and contributed to the first PC variable (*PC1LARS*) in the PCA. Others, including liquid stool incontinence and flatus incontinence, accounted for a greater proportion of the second PC variable (*PC2LARS*) (Fig. 1).

In addition, the sum of scores for items regarding frequency, clustering, and urgency was referred to as *sub1LARS*, while the sum of scores for incontinence for flatus and liquid stools was referred to as *sub2LARS*. Therefore, similar to the previous research^33^, the *PC1LARS* and *sub1LARS* corresponded to the frequency-dominant LARS, and the *PC2LARS* and *sub2LARS* corresponded to the incontinence-dominant LARS.

Table 1 summarizes the description of these LARS-associated variables and the range of each variable. For the *totalLARS*, pre-defined indicators of LARS questionnaires were applied. To further interpret the new symptom-based LARS subgroups, we introduced grouping criteria for each LARS-associated variable. In the case of subgrouping items in the LARS questionnaires, binary group variables derived from the new continuous variables were devised. The criteria applied to each level are shown in the "levels" column of this table. Supplementary Table S2 summarizes the distribution of the LARS-associated variables.

**Table 1:** Low anterior resection syndrome (LARS)-associated variables.

LARS-associated continuous variables					LARS-associated binary variables		
Variable name	Description	Range			Variable name	Levels	N
		Min	Mean	Max			
*totalLARS*	The sum of the scores for all five answers in LARS questionnaire	24	35.24	41	*totalLARS_group*	Major: total LARS ≥ 30	91
						Minor: total LARS ≤ 29	18
*PC1LARS* (*frequency-dominant*)	Principle component 1 in PCA with each score for five LARS answers	−4.003	0	1.468	–		
*PC2LARS* (*incontinence-dominant*)	Principle component 2 in PCA with each score for five LARS answers	−2.575	0	2.097	–		
*sub1LARS* (*frequency-dominant*)	The sum of the scores for the 3rd, 4th, and 5th answers in LARS questionnaire	15	28.48	32	*sub1LARS_group*	Severe: sub1LARS ≥ 29	78
						Mild: sub1LARS ≤ 28	31
*sub2LARS* (*incontinence-dominant*)	The sum of the scores for the 1st and 2nd answers in LARS questionnaire	0	6.761	10	sub2LARS_group	Severe: sub2LARS ≥ 7	76
						Mild: sub2LARS ≤ 6	33

*PCA* principal component analysis.

The cutoff value of LARS-associated binary variables was determined by considering the distribution of patients. In the *sub1LARS*, the “severe” classification was determined when bowel frequency was more than four times a day together with clustering symptom and urgency at least once a week. In the *sub2LARS*, “severe” refers to symptoms of incontinence for both flatus and liquid stools that occur at least once per week (Supplementary Table S3). The two group factors were significantly independent of each other (chi-square = 0.410, P = 0.522).

### Microbial diversity

Microbial alpha diversity was measured using different methods for richness and diversity in order to compare the LARS groups (Table 2). No significant correlation was noted between the *totalLARS* score and all alpha-diversity indices, whereas a significant correlation was noted between the *sub1LARS* score or *sub2LARS* and alpha diversity richness. Briefly, the microbial richness index, including Observed feature number and Chao1, was monitored, revealing a significantly negative correlation in *sub1LARS* [Spearman correlation coefficient (ρ) = −0.217, P = 0.023 with Observed feature number; ρ = −0.222, P = 0.020 with Chao1], while showing a significantly positive correlation in *sub2LARS* (ρ = 0.221, P = 0.021 with Observed feature number; ρ = 0.221, P = 0.021 with Chao1).

**Table 2:** Pair-wise correlation coefficients between the low anterior resection syndrome associated group variables (continuous) and microbial alpha-diversity indices.

	Pearson coefficient				Spearman coefficient			
	Observed	Chao1	Shannon	InvSimpson	Observed	Chao1	Shannon	InvSimpson
*totalLARS*	−0.024	−0.026	−0.033	−0.06	0.035	0.032	−0.008	−0.033
*PC1LARS*	−0.235*	−0.236*	−0.226*	−0.235*	−0.267 **	−0.272 **	−0.241*	−0.214*
*PC2LARS*	0.124	0.125	0.069	0.028	0.172	0.174	0.050	−0.010
*sub1LARS*	−0.191*	−0.193*	−0.17	−0.162	−0.217 *	−0.222 *	−0.189*	−0.177
*sub2LARS*	0.153	0.152	0.119	0.075	0.221*	0.221*	0.128	0.076

*P < 0.05, **P < 0.01.

**Figure 2:**
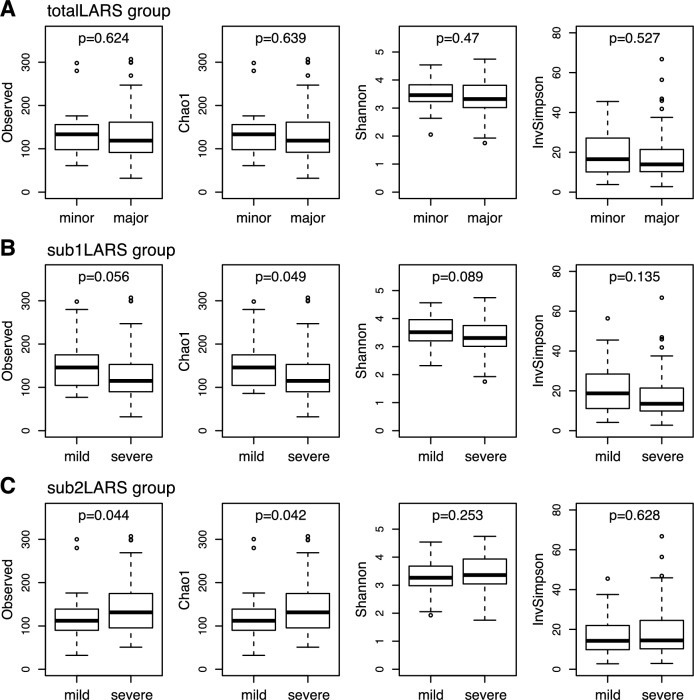
Comparison of microbial alpha-diversity between low anterior resection syndrome (LARS) groups. (**A**) *totalLARS_group*, (**B**) *sub1LARS_group*, (**C**) *sub2LARS_group*. Wilcoxon rank-sum tests were performed in each comparison, and nominal P-values are shown.

Further, a mean comparison analysis of the alpha diversity indices for each LARS-associated binary variable was performed (Fig. 2). The microbial richness, assessed using Chao1, was significantly lower in the severe level of *sub1LARS_group* [median 115 (SD 59.1) vs. 146 (57.7), severe vs. mild, respectively, P = 0.0487] but higher in the severe level of *sub2LARS_group* [132 (60.1) vs. 112 (55.5), P = 0.0419]. Beta-diversity showed no difference between LARS groups (Supplementary Fig. S1).

### Severity of LARS according to enterotypes of the gut microbiome

**Figure 3:**
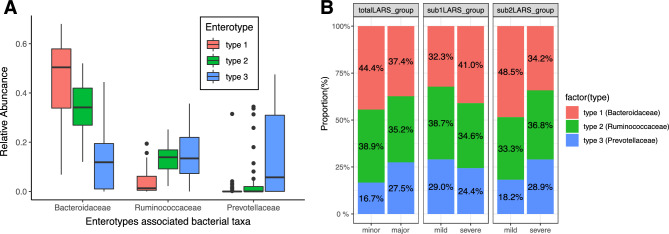
Enterotypes of gut microbiome according to severity and groups with low anterior resection syndrome (LARS). (**A**) Relative abundance of main contributing family-level taxa for three enterotypes of the gut microbiome. (**B**) Composition of three enterotypes among LARS groups. The values shown on the bar graph (**B**) represent the proportion of each enterotype in the column (levels) at the LARS group. The enterotype proportion at each column under each LARS group is shown as the percentage value in the bar graph. No significant difference in the enterotype composition between minor/mild and major/severe in each LARS group (Chi-squared test, P > 0.05).

The gut microbiome was clustered into three enterotypes, to which the main bacterial taxa contributing were *Prevotellaceae*, *Ruminococcaceae*, and *Bacteroidaceae* at the Family level (Fig. 3a). A different composition was identified in the *sub1LARS_group* compared with *sub2LARS_*group and *totalLARS_group* (Fig. 3b). In the severe *sub1LARS_group,* the proportion with *Bacteroidaceae* enterotype was higher, and those with *Prevotellaceae* enterotype tended to be lower than those in the mild *sub1LARS_group* (P = 0.692). In contrast, *sub2LARS_group* and *totalLARS_group* showed higher *Prevotellaceae* enterotype in the severe group than in the mild group (P = 0.625 and P = 0.311, respectively).

### Differentially abundant taxa between LARS groups

**Figure 4:**
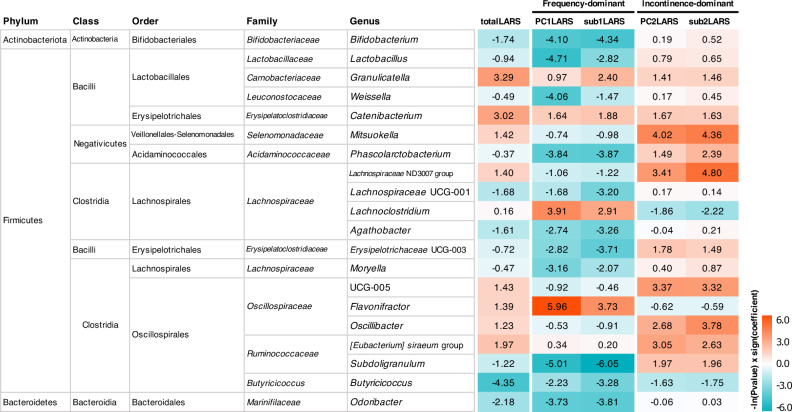
Heatmap of relationship between bacterial taxonomy and severity of low anterior resection syndrome (LARS) by LARS groups. Twenty taxa with any significant result (nominal P-value) corresponding to the severity of LARS are listed at the genus level. Log-transformed P values with sign of coefficient for each MaAsLin2 linear model are shown on the heatmap. Genera with zero in over 90% of samples were excluded for the analysis and a total of 123 genera were used. Each taxon with a positive relationship with LARS severity is marked red, while those with a negative relationship are marked blue.

Twenty genera among a total of 123 genera showed a significant relationship (the absolute value of Spearman’s rank coefficient was above 0.2, nominal P < 0.05) to at least one variable among the LARS-associated continuous variables (Fig. 4).

As the *totalLARS* score increased, the abundance of *Granulicatella* (linear model coefficient β = 0.27; 95% CI, 0.02 to 0.52; P = 0.037) and *Catenibacterium* (β = 0.26; 95% CI, 0 to 0.51; P = 0.049) increased, and the abundance of *Butyricicoccus* (β = −0.55; 95% CI, −0.97 to −0.12; P = 0.013) decreased, and the direction of the relationship was consistent with other LARS-associated variables.

*PC1LARS* and *sub1LARS* or *PC2LARS* and *sub2ALRS* showed similar correlation patterns for each genus, whereas each distinct symptom groups, frequency-dominant vs. incontinence-dominant, differed significantly in the relationship with taxa. Main genera of lactic acid-producing bacteria, *Bifidobacterium* (β = −0.76; 95% CI, −1.37 to −0.15; P = 0.016 with *PC1LARS* and β = −0.79; 95% CI, −1.40 to −0.18; P = 0.013 with *sub1LARS*), *Lactobacillus* (β = −0.58; 95% CI, −1.00 to −0.15; P = 0.009 with *PC1LARS* and β = −0.42; 95% CI, −0.85 to 0.01; P = 0.060 with *sub1LARS*), and *Weissella* (β = −0.33; 95% CI, −0.60 to −0.06; P = 0.017 with *PC1LARS*) showed a significantly negative relationship with the severity of the frequency-dominant pattern, while showing a weak positive relationship with the severity of the incontinence-dominant patterns (*PC2LARS* and *sub2LARS*).

**Figure 5:**
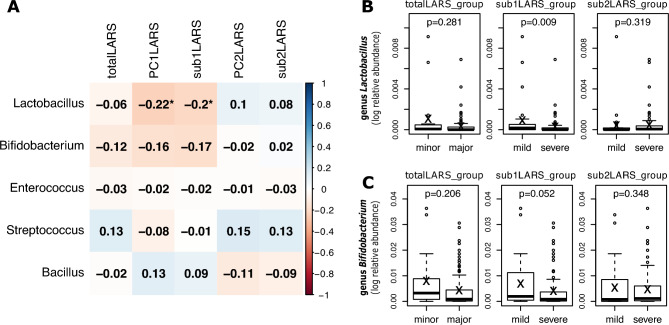
Abundancy differences of lactic acid-producing microbiome between low anterior resection syndrome (LARS) groups. (**A**) Spearman correlation between LARS severity in LARS groups and the relative abundance of microbiome (*P < 0.05). (**B,C**) Comparison of the relative abundance of genus *Lactobacillus* and *Bifidobacterium* in minor (mild) and major (severe) LARS groups (P values of MaAsLin2 linear model are shown).

The differential abundance analysis with binary variables was performed. The abundance difference of *Butyricicoccus* in *totalLARS_group* (β = −1.34; 95% CI, −2.48 to −0.2; P = 0.023), *Subdoligranulum* in *sub1LARS_group* (β = −2.52; 95% CI, −4.07 to −0.96; P = 0.002), and *Oscillibacter* in *sub2LARS_group* (β = 1.74; 95% CI, 0.38 to 3.10; P = 0.014) was significant. The abundance of *Lactobacillus* (β = –1.28; 95% CI, −2.21 to −0.34; P = 0.009) and *Bifidobacterium* (β = −1.36; 95% CI, −2.73 to 0; P = 0.052) was lower in the severe *sub1LARS_group* than in the mild group (Fig. 5).

### Abundance of probiotic strains among LARS severity

*Lactobacillus, Streptococcus, Enterococcus, Bacillus,* and *Bifidobacterium* are used as probiotics^38^. *PC1LARS* and *sub1LARS* had a significant negative correlation with *Lactobacillus* [Spearman correlation coefficient [ρ] = −0.223, P = 0.020; ρ = −0.205, P = 0.033, respectively, Fig. 5a]. In *sub1LARS_group*, severe group showed statistically lower *Lactobatillus* (P = 0.009, Fig. 5b) and lower *Bifidobacterium* (P = 0.052, Fig. 5c).

## Discussion

This study is the first to reveal the gut microbiome of LARS patients and to identify taxonomic differences in relation to dominant symptoms. As reported previously^33^, the main symptoms of LARS were clustered into two groups: frequency-dominant and incontinence-dominant. In the LARS questionnaire, frequency-dominant symptoms were expressed through questions regarding clustering, urgency, and frequent bowel movements, whereas incontinence-dominant symptoms were expressed through fecal and flatus incontinence. The questions were classified into two groups, and some taxa of the gut microbiome were differentially expressed according to the two dominant LARS symptom groups. The *Prevotellaceae* enterotype and lactic acid producers, including *Lactobacillus* and *Bifidobacterium*, were at lower levels in the *sub1LARS_group* with severe frequency, clustering, and urgency.

Severe *sub1LARS_group* had lower alpha diversity compared to mild *sub1LARS_group*. High richness and diversity of the gut microbiome are considered characteristics of a healthy gut microbial ecosystem, which reflects the stability and resilience of the microbiome. Vandeputte et al. reported that loose stool consistency, in relation to rapid transit time, was associated with reduced observed species richness, which reached a minimum in diarrhea-afflicted individuals^39^. Tian et al. reported that transit time had a significant association with diversity indices^40^. Frequent bowel movements in patients with LARS may result in low gut microbial diversity.

*Sub1LARS_group* had a distinctive taxonomic composition. The frequent bowel movements and evacuatory dysfunctions in LARS patients may be associated with the gut microbiome. Therefore, modification of the microbiome could adjust these dysfunctions. The autonomic and enteric nervous systems control the bowel motility of the distal colon and rectum, while the autonomic nerve fibers to the distal colon, which becomes the neorectum after LAR, are transected^11^. Due to the destruction of an inhibitory alpha-sympathetic pathway, extrinsic denervation results in increased motility, which may account for the frequent bowel movements observed in LARS patients^41^. The enteric nervous system subsequently controls the neorectum, and serotonin (5-HT) receptors allow intrinsic primary afferent neurons to sense and regulate the enteric nervous system. 5-HT receptor activity can be modulated by the serotonin receptor antagonist ramosetron, commonly prescribed to LARS patients^42^; furthermore, LARS may also be regulated by the gut microbiome due to its previous reported ability to modify the levels of 5-HT in the colon^19,21^.

Some studies have reported symptom improvement in LARS patients using probiotics. However, they had several limitations. A randomized controlled trial (RCT) administered probiotics during 4 weeks to LARS patients in order to improve bowel function following an ileostomy closure but failed to demonstrate any improvement in the postoperative GI QOL index of patients^15^. Another RCT using a single-strain probiotic, *Lactobacillus plantarum* DJLP243, also failed to demonstrate significant effects on bowel dysfunction following ileostomy reversal^14^. These studies were conducted during the perioperative period of ileostomy reversal when patients experienced the most severe intestinal dysfunction following stoma restoration, and gut microbiome changes were not analyzed. Our study included only patients who completed acute cancer care, and their gut microbiome was comprehensively analyzed to understand the relationships between the gut microbiome and their symptoms.

Although the aforementioned RCTs did not demonstrate significant improvements in bowel dysfunction after ileostomy reversal, these studies were initiated based on the hypothesis that the gut microbiome is related to bowel motility and GI functional disorders, which was supported by previous studies^18–21^. 5-HT (a key regulator of GI motility) levels in the colon epithelium and lumen are influenced by short-chain fatty acids produced by gut microbiota via tryptophan hydroxylase 1 in enterochromaffin cells in the lining of the GI tract^18,21^. Neuronal reprogramming is an additional hypothesis explaining the relationship between the gut microbiome and GI function^19,20^. Therefore, unveiling the gut microbiome of LARS patients will help develop ways to improve LARS.

This study was the first to comprehensively analyze the gut microbiome of patients at the diversity, enterotype, and species levels and to compare microbiome taxa according to predominant symptom-based LARS groups. Patients with incontinence-dominant LARS (*sub2LARS_group*) may have an injury to the internal sphincter; therefore, the improvement of the symptoms by means of the modulation of gut mobility with probiotics is unlikely. However, the frequency-dominant LARS (*sub1LARS_group*) exhibited decreased alpha diversity and low number of lactic acid-producing bacteria, including *Lactobacillus* and *Bifidobacterium*, which are commonly produced as probiotics, suggesting that probiotic induced microbiome modulation may benefit the *sub1LARS_group* to alleviate LARS symptoms. This microbiome analysis study may serve as the basis for future microbiome modulation studies specifically targeting the *sub1LARS_group*.

This study has some limitations. First, postoperative dietary changes might influence the gut microbiome composition because patients attempt to adapt to LARS-associated symptoms. Based on past experiences, individuals with severe frequency-related symptoms will attempt to consume foods that alleviate symptoms. Moreover, such food differences among groups might influence the gut microbiome, although no dietary information was collected in this study. Second, whether the microbiome in the gut was the cause of the LARS symptoms was unclear. As this was an observational study and not an interventional one, we were unable to explain the causality of the results. Third, we included only patients with LARS symptoms. The patients in this study were almost male with low-lying rectal cancer who received radiotherapy pre or postoperatively. Therefore, the patients might have deviated characteristics from the standard group of rectal cancer patients, which could bias the results. Because we aimed to elucidate the relationship between LARS symptoms and microbiome, we only included patients with LARS symptoms. To know the sole effect of microbiome on LARS considering the other interference of host and treatment related factors, we need further research.

In conclusion, LARS was classified into frequency-dominant and incontinence-dominant LARS groups. Patients with severe frequency-dominant LARS had decreased microbial diversity and exhibited a higher *Bacteroidaceae* enterotype, a lower *Prevotellaceae* enterotype, and fewer lactic acid-producing microorganisms, including *Bifidobacterium* and *Lactobacillus* than the non-severe frequency-dominant LARS patients, which was not observed in the incontinence-dominant LARS group. Future studies are needed to evaluate the effects of *Lactobacillus* and/or *Bifidobacterium* in patients with frequency-dominant LARS to monitor the alleviation of LARS symptoms.

## Supplementary Information

Supplementary Information.

## Data Availability

The raw sequencing data generated and analysed during the current study are available in the Sequence Read Archive (SRA) repository (SRX17679639–SRX17679747) under BioProject PRJNA882613.
