# Supplementary material for: Gut microbiome associated with low anterior resection syndrome after rectal cancer surgery
Source: Sci Rep. 2023 May 26;13:8578. doi: 10.1038/s41598-023-34557-2 (PMC10220038; doi:10.1038/s41598-023-34557-2)
Supplement: Supplementary file 1 — Supplementary Information. [file 41598_2023_34557_MOESM1_ESM.docx]

Supplementary information

**Gut Microbiome Associated with Low Anterior Resection Syndrome after Rectal Cancer Surgery**

Soyoung Park, Ph.D.^1^, Minjung Kim, M.D.^2,3,4*^, Ji Won Park, M.D., Ph.D.^2,3,4^, Jinsun Choi, M.D.^2^, Hyo Jun Kim, M.D.^2^, Han-Ki Lim, M.D.^2,3^, Seung-Bum Ryoo, M.D., Ph.D.^2,3^, Kyu Joo Park, M.D., Ph.D.^2,3^, Yosep Ji, Ph.D.^1^, and Seung-Yong Jeong, M.D., Ph.D.^2,3,4^

^1^Bioinformatics Center, HEMpharma, Suwon-si, Gyeonggi-do, Republic of Korea

^2^Department of Surgery, Seoul National University College of Medicine, Seoul, Republic of Korea

^3^Colorectal Cancer Center, Seoul National University Cancer Hospital, Seoul, Republic of Korea

^4^Cancer Research Institute, Seoul National University, Seoul, Republic of Korea

***Corresponding author:** Minjung Kim, M.D.

Department of Surgery, Seoul National University College of Medicine, Seoul, Republic of Korea

101, Daehak-ro Jongno-gu, Seoul 03080, Republic of Korea

Tel: +82-2-2072-1272

Fax: +82-2-766-3975

E-mail: minjungkim@snuh.org

Supplementary Figure S11. Microbial beta-diversity of 109 low anterior resection syndrome (LARS) patients colored by each LARS group. Principal coordinated analysis (PCoA) was performed with Bray-Curtis distance (A) and Jaccard distance (B) between samples after collapsing bacterial features at the genus level. R^2^ and P value from Permutational multivariate ANOVA (PERMANOVA) test are shown.


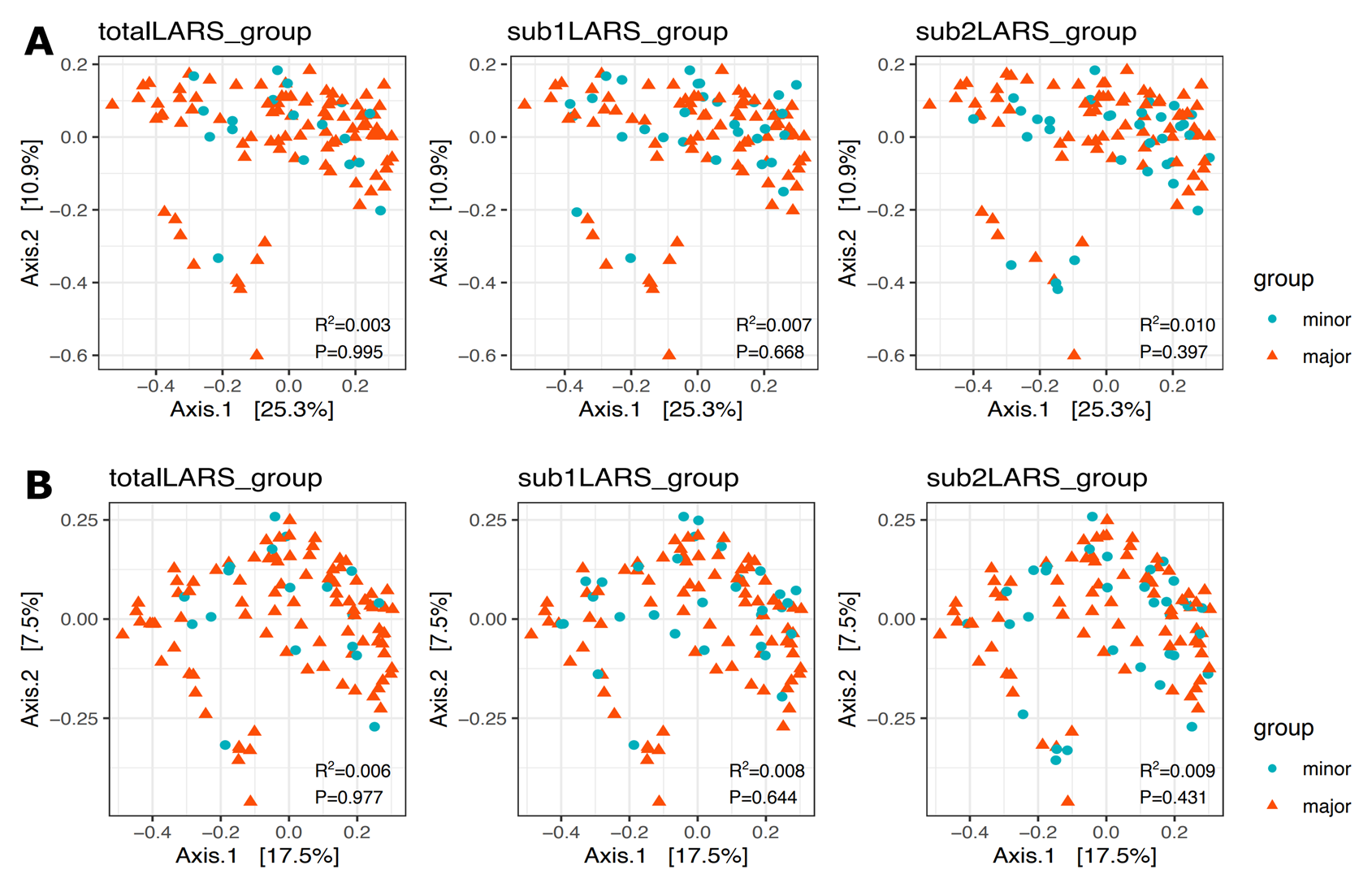


**Supplementary Table S1. Baseline characteristics of patients with low anterior resection syndrome**

| Variables | n=109 |
| --- | --- |
| Mean age, year (±SD) | 62.2 (±9.7) |
| Sex (%) |  |
| Male | 101 (92.7) |
| Female | 8 (7.3) |
| ASA classification (%) |  |
| I | 31 (28.4) |
| II | 73 (67.0) |
| III | 5 (4.6) |
| Mean CEA, ng/mL (range) | 4.3 (0.4–52.9) |
| Tumor location from the anal verge |  |
| Mean, cm (±SD) | 7 (5.0) |
| Missing | 6 |
| Operative time |  |
| Mean, min (±SD) | 175.4 (±96.4) |
| Missing | 4 |
| Diverting ileostomy (%) | 64 (58.7) |
| Postoperative complication (%) | 36 (33.0) |
| Anastomosis leakage (%) | 3 (2.8) |
| T stage (%) |  |
| T0/is | 10 (9.2) |
| T1 | 17 (15.6) |
| T2 | 26 (23.8) |
| T3 | 51 (46.8) |
| T4 | 5 (4.6) |
| N stage (%) |  |
| N0 | 66 (60.6) |
| N1 | 35 (32.1) |
| N2 | 8 (7.3) |
| Preoperative or postoperative radiotherapy (%) | 55 (50.5) |
| Adjuvant chemotherapy (%) | 45 (41.3) |

SD, standard deviation, ASA, the American Society of Anesthesiologist, CEA, carcinoembryonic antigen

**Supplementary Table S2. Distribution of 109 patient data according to score combinations for each LARS subgroup.** Possible score-combinations were listed by LARS subgroup (Q1+Q2 for sub2LARS or Q3+Q4+Q5 for sub1LARS) and sample number (count) and cumulated percent by each combination from 109 patients’ data were shown.

| **Summation of Q1 and Q2 (*sub2LARS*)** | | | | |  | data | |
| --- | --- | --- | --- | --- | --- | --- | --- |
|  | Incontinence for flatus (0,4,7) | Incontinence for liquid stools (0,3) |  | Score |  | Count | Cumulated percent (%) |
| Possible score-combinations   of incontinent-based items | 0 | 0 |  | 0 |  | 12 | 11.0 |
|  | 0 | 3 |  | 3 |  | 11 | 21.1 |
|  | 4 | 0 |  | 4 |  | 10 | 30.3 |
|  | 4 | 3 |  | 7 |  | 17 | 45.9 |
|  | 7 | 0 |  | 7 |  | 15 | 59.6 |
|  | 7 | 3 |  | 10 |  | 44 | 100.0 |
| **Summation of Q3, Q4 and Q5 (*sub1LARS*)** | | | | |  | Data | |
|  | Frequency (0,2,4,5) | Clustering (0,9,11) | Urgency (0,11,16) | Score |  | Count | Cumulated percent (%) |
| Possible score-combinations   of frequency-based items | 4 | 11 | 0 | 15 |  | 1 | 0.9 |
|  | 0 | 9 | 11 | 20 |  | 3 | 3.7 |
|  | 0 | 11 | 11 | 22 |  | 2 | 5.5 |
|  | 2 | 9 | 11 | 22 |  | 3 | 8.3 |
|  | 2 | 11 | 11 | 24 |  | 9 | 16.5 |
|  | 0 | 9 | 16 | 25 |  | 1 | 17.4 |
|  | 4 | 11 | 11 | 26 |  | 5 | 22.0 |
|  | 0 | 11 | 16 | 27 |  | 5 | 26.6 |
|  | 2 | 9 | 16 | 27 |  | 2 | 28.4 |
|  | 2 | 11 | 16 | 29 |  | 30 | 56.0 |
|  | 4 | 9 | 16 | 29 |  | 1 | 56.9 |
|  | 4 | 11 | 16 | 31 |  | 44 | 97.2 |
|  | 5 | 11 | 16 | 32 |  | 3 | 100.0 |
| Total |  |  |  |  |  | 109 |  |

**Supplementary Table S3. Contingency table of 109 patients according to each LARS subgroup.** Sample count across two LARS subgroups (sub1LARS_group and sub2LARS_group) are shown as "Count," and the expected count and ratio of each cell are also listed. Two group factors are statistically independent (p-value = 0.522 from a chi-square test).

|  |  |  | ***sub1LARS***  **(Frequency-dominant  LARS pattern)** | | Row Total |
| --- | --- | --- | --- | --- | --- |
|  |  |  | Mild | Severe |  |
| ***sub2LARS***  **(Incontinence-dominant LARS pattern)** | Mild | Count | 8 | 25 | 33 |
|  |  | Expected Count | 9.385 | 23.615 |  |
|  |  | Count / Row Total | 0.242 | 0.758 | 0.303 |
|  |  | Count/ Col Total | 0.258 | 0.321 |  |
|  |  | Count/ Table Total | 0.073 | 0.2289 |  |
|  | Severe | Count | 23 | 53 | 76 |
|  |  | Expected Count | 21.615 | 54.385 |  |
|  |  | Count / Row Total | 0.303 | 0.697 | 0.697 |
|  |  | Count/ Col Total | 0.742 | 0.679 |  |
|  |  | Count/ Table Total | 0.211 | 0486 |  |
| Column Total | | | 31 | 78 | 109 |
|  |  |  | 0.284 | 0.716 |  |

LARS, low anterior resection syndrome
